# Aescin Protects Neuron from Ischemia-Reperfusion Injury via Regulating the PRAS40/mTOR Signaling Pathway

**DOI:** 10.1155/2020/7815325

**Published:** 2020-09-30

**Authors:** Xinjie Gao, Heng Yang, Jiabin Su, Weiping Xiao, Wei Ni, Yuxiang Gu

**Affiliations:** ^1^Department of Neurosurgery, Huashan Hospital, Fudan University, Shanghai 200040, China; ^2^Neurosurgical Institute, Fudan University, Shanghai 200040, China; ^3^Shanghai Clinical Medical Center of Neurosurgery, Shanghai 200040, China; ^4^Shanghai Key Laboratory of Brain Function and Restoration and Neural Regeneration, Shanghai 200040, China

## Abstract

Ischemic stroke is one of the major causes of disability; widely use of endovascular thrombectomy or intravenous thrombolysis leads to more attention on ischemia-reperfusion injury (I/R injury). Aescin, a natural compound isolated from the seed of the horse chestnut, has been demonstrated anti-inflammatory and antiedematous effects previously. This study was aimed at determining whether aescin could induce protective effects against ischemia-reperfusion injury and exploring the underlying mechanisms in vitro. Primary cultured neurons were subjected to 2 hours of oxygen-glucose deprivation (OGD) followed by 24 hours of simulated reperfusion. Aescin, which worked in a dose-dependent manner, could significantly attenuate neuronal death and reduce lactate dehydrogenase (LDH) release after OGD and simulated reperfusion. Aescin treatment at a concentration of 50 *μ*g/ml provided protection with fewer side effects. Results showed that aescin upregulated the phosphorylation level of PRAS40 and proteins in the mTOR signaling pathway, including S6K and 4E-BP1. However, PRAS40 knockdown or rapamycin treatment was able to undermine and even abolish the protective effects of aescin; meanwhile, the levels of phosphorylation PRAS40 and proteins in the mTOR signaling pathway were obviously decreased. Hence, our study demonstrated that aescin provided neuronal protective effects against I/R injury through the PRAS40/mTOR signaling pathway in vitro. These results might contribute to the potential clinical application of aescin and provide a therapeutic target on subsequent cerebral I/R injury.

## 1. Introduction

Stroke is a major cause of disability globally; 85% are ischemic and responsible for approximately 5 million deaths every year [1, 2]. The commonest cause of ischemic stroke is pathophysiological thrombosis or thromboembolism giving rise to cerebral arterial occlusion [3]. Nowadays, endovascular thrombectomy and intravenous thrombolysis have been widely recommended and performed to treat patients with acute ischemic stroke. However, not all patients end up with good outcomes after endovascular thrombectomy or intravenous thrombolysis. Apart from some known complications, such as intraoperative hemorrhage, subsequent ischemia-reperfusion injury (I/R injury) may be one of the most important factors contributing to the poor prognosis [4]. Owing to that I/R injury is a series of complex pathophysiological mechanisms and involves various signaling pathways [5–8], till now, no effective measures have been developed for cerebral I/R injury in a clinic.

Aescin is a natural compound isolated from the seed of the horse chestnut [9]. Studies have demonstrated the anti-inflammatory, antiedematous, and neuroprotective effects of aescin. It is reported that aescin treatments could provide a neuroprotective effect via inhibiting cell apoptosis [10]. Also, it is capable to inhibit the increase of capillary permeability, attenuate cerebral edema, and reduce the adhesiveness and migration of neutrophils induced by various factors [11–13]. To date, the majority of researches about aescin stated the application on cerebral trauma and intracranial hemorrhage. Nevertheless, limited comprehensive studies have been carried out in vitro or in vivo to address whether it can be applied to administrate subsequent cerebral I/R injury.

Accumulating evidences suggest that the neurobiological function of aescin is related to the Akt and mammalian target of rapamycin (mTOR) signaling pathway [14, 15]. For instance, aescin protected retinal pigment epithelium cells from oxidative stress via activating the Akt signaling pathway [16]. In Huntington's disease, it promoted neurodegeneration through mTOR signaling pathways [15]. Our previous studies found that the mTOR signaling pathway played an important role in the protection against brain I/R injury both in vitro and in vivo [17, 18]. Additionally, the proline-rich Akt substrate of 40 kDa (PRAS40) played a pivotal role in protecting against I/R injury by linking the Akt and mTOR pathways [19]. According to all of these findings, we supposed that there was a potential association between aescin and the PRAS40/mTOR pathway.

In this study, we used the oxygen-glucose deprivation (OGD) and reperfusion model in primary cultured neurons to simulate the neuronal I/R injury in vitro and examined whether aescin treatment could alleviate neuronal I/R injury. What is more, we also try to figure out whether the neuroprotective effects of aescin are mediated by regulating the PRAS40/mTOR signaling pathway.

## 2. Materials and Methods

### 2.1. Animals

All pregnant C57BL/6 mice were purchased from Slac Laboratory Animal Co. (Shanghai, China) and maintained in the animal facility at Fudan University. All experimental protocols were approved by the Fudan University Department of Laboratory Animal Science. Mice were allowed free access to food and water before all procedures.

### 2.2. Primary Neuronal Cultures

The methods for the culture of well-characterized embryonic mice primary cortical neurons are described in detail in published works [20]. Timed-pregnant C57BL/6 mice (E16-17) were euthanized with isoflurane, and the embryos were removed from the uterus. The scalp and skull were cut. The cerebral cortex was freshly dissected and placed in a dish containing cold DMEM/F12 (Gibco) culture medium without calcium and magnesium. Meninges and blood vessels were carefully peeled off under a microscope. Then, the cortices were cut into pieces, digested in papain for 10 min at 37°C, and shaken two to three times by Pasteur pipettes. Complete media containing 10% fetal bovine serum were added to terminate digestion. Tissues were pipetted about 10 times slightly using the sterile Pasteur pipettes. Cell suspension was allowed to stand for 2 min, collected in a new 50 ml centrifuge tube, and filtered with a 40 *μ*m cell strainer. Repeat the above step twice. Then, cell suspension was centrifuged at 1000 rpm/min at 4°C for 5 min. The supernatant was discarded. Cells were resuspended in the DMEM/F12 culture medium containing 10% fetal bovine serum. The cell resuspension was stained using trypan blue, and cells were counted using a hemocytometer under a microscope. Cells were inoculated at a density of 2 × 10^5^ cells/cm^2^ into a 6-well plate or a 96-well plate precoated with poly-D-lysine. After 1 hour, the medium was completely switched to Neurobasal™ medium (Gibco) supplemented with 1% GlutaMAX™ (Gibco) and 2% B-27 (Gibco). Half medium was changed every 3 days. Cultures were incubated at 37°C in a 5% CO_2_ incubator, and experiments were performed 8 days after incubation.

### 2.3. In Vitro Lentivirus Gene Transfer

The lentivirus, containing PRAS40 (PRAS40 group) or PRAS40 shRNA (PRAS40 KD group), diluted with PBS buffer to 1 *μ*l for 96-well plates and to 10 *μ*l for 6-well plates, was directly added into the medium of 4-day primary cultured neurons with the multiplicity of infection (MOI) at 3. The same amount of vector (vector group) was also added to the medium as an experimental control. Then, cells were incubated at 37°C in a 5% CO_2_ incubator for another 3 days before OGD.

### 2.4. In Vitro Oxygen-Glucose Deprivation (OGD) and Reperfusion Model

Primary cultured neurons were plated into 96-well culture plates or 6-well plates at a density of 2 × 10^5^ cells/cm^2^. Then, cells were washed twice with glucose-free artificial cerebrospinal fluid (ACSF), and the plates were transferred to a modular hypoxic chamber (MIC-101, Billups-Rothenberg, Del Mar, CA) filled with mixed gases of 5% CO_2_ and 95% N_2_. The oxygen level was maintained at less than 0.02% at 37°C. The cells were kept in the hypoxic chamber for 2 hours. Neurons were then restored with a complete neurobasal medium containing 1% GlutaMAX™ and 2% B-27 and recovered at normoxic conditions (37°C, 5% CO_2_) for 24 hours to simulated reperfusion in vitro. OGD samples without any treatments were defined as the control group (Con group). The no-OGD groups (sham group) were washed twice with 10 mM glucose in ACSF without oxygen deprivation.

### 2.5. Aescin Treatments and Rapamycin Pretreatment

Aescin, obtained from Luye Pharma (H2000323, Shanghai, China), was dissolved in normal saline (NS). Aescin was added at the beginning of simulated reperfusion (aescin group), and the final concentrations were administrated ranging from 0 to 100 *μ*g/ml in the complete medium. Normal saline at the same volume was added as a reference (NS group). Rapamycin was obtained from Sigma-Aldrich (V900930, St. Louis, MO, USA) and was dissolved in dimethyl sulfoxide (DMSO). Cells on culture plates were treated with rapamycin (Rapa group) at a final concentration of 100 nM at the beginning of simulated reperfusion simultaneously.

### 2.6. Cell Viability Assay

As previously described, cell viability was quantified by measuring lactate dehydrogenase (LDH) release at 24 hours of reperfusion after OGD for 2 hours using the Cytotoxicity Detection Kit (Roche Applied Science) [21]. Briefly, the incubation buffer harvested from the 96-well plate 24 hours after reperfusion was centrifuged at 12,000 rpm for 15 min; then, 100 ml of the cell-free supernatant was transferred to a new 96-well plate. The supernatant was incubated with the reaction mixture from the kit for 30 min. LDH activity was determined via a colorimetric assay (Bio-Rad Laboratories, Hercules, CA). After the removal of the incubation buffer from a 96-well plate, 1% Triton X-100 lysing solution was applied to the remaining cells. The percentage of LDH released to the incubation buffer compared to total LDH was calculated as follows:

(1) $$\begin{array}{c} \text{LDH release ratio}=\frac{\text{released LDH in buffe}}{\text{released LDH in buffer}+\text{intracellular LDH }}. \end{array}$$

### 2.7. Protein Preparation and Western Blotting

To investigate the effects of aescin on the protein expression of the PRAS40/mTOR signaling pathway after OGD and reperfusion, whole-cell protein was extracted and western blot was performed as described before [22]. Briefly, primary cultured neurons grown in 6-well plates were harvested 24 hours after OGD and reperfusion and homogenized in cold RIPA buffer (9806; CST, Danvers, MA, USA), containing 1 mmol/l PMSF and a protease inhibitor cocktail (1 : 20, Cat# P-2714; Sigma, St. Louis, MO, USA). Simultaneously, the protein of the control group was also prepared for western blot. The homogenate was centrifuged at 12000 rpm for 15 min at 4°C, and the supernatant was collected for protein detection. 25 *μ*g of protein was loaded into each lane and subjected to SDS-PAGE using 4%-15% Ready Gel (Catalog #L050505A2; Bio-Rad, Hercules, CA, USA) at 120 V for 120 min. Protein bands were transferred to polyvinylidene fluoride membranes (Millipore, Bedford, MA, USA) at 250 mA for 3.5 hours. Membranes were incubated overnight with primary antibodies at 4°C followed by HRP-labeled secondary antibody (Invitrogen, Eugene, OR, USA) for 1 hour at room temperature. All the primary antibodies used are listed in Table 1. Membranes were scanned using Typhoon Trio (GE Healthcare, Amersham, Buckinghamshire, UK). Optical densities of all protein bands were analyzed using the IMAGEQUANT 5.2 software (GE Healthcare).

### 2.8. Statistical Analyses

The GraphPad Prism 8.0 software (GraphPad Software, La Jolla, CA, USA) was used for statistical analyses. Student's *t*-tests were used when 2 groups were compared. One-way or two-way Analysis of Variance (ANOVA) was used for multiple comparisons followed by the post hoc test. Differences were considered statistically significant at *P* values < 0.05. All data are expressed as mean ± SEM.

## 3. Results

### 3.1. Aescin Provided Protective Effects of Neuron against I/R Injury In Vitro

**Figure 1:**
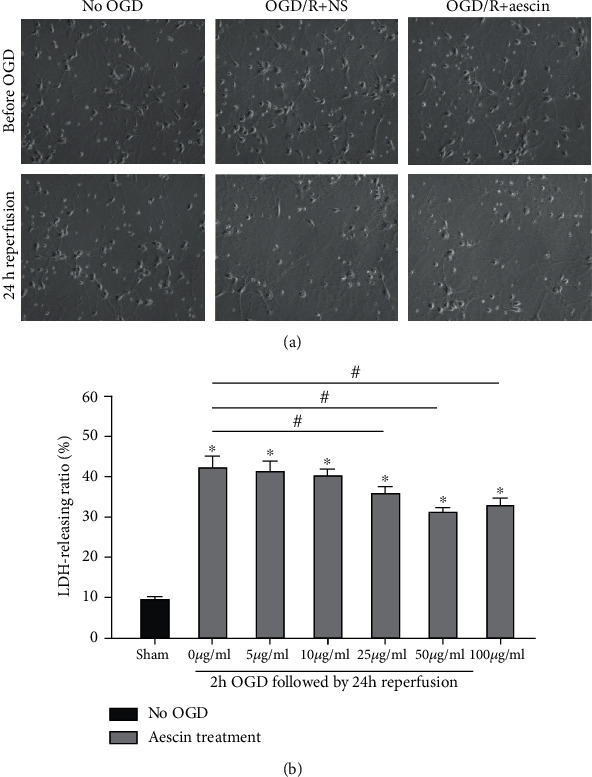
Neuroprotective effects of aescin treatment in vivo. (a) Primary neurons matured after 7 days from isolation. Neurons were subjected to 2 hours of OGD followed by 24 hours of reperfusion to simulate I/R injury in vitro. Neurons treated with aescin showed less axonotmesis compared with the OGD group. (b) LDH release was measured 24 hours after the experiments. All data after OGD and reperfusion were normalized to the values of the sham group (no OGD). After 2 hours of OGD and 24 hours of reperfusion, the LDH release ratio was significantly increased compared to the sham group. Aescin was administrated at various concentrations (5-100 *μ*g/ml); treatments at concentrations of 25 *μ*g/ml, 50 *μ*g/ml, and 100 *μ*g/ml aescin had protective effects in the form of reducing the LDH release induced by OGD and reperfusion compared with the no aescin treatment group (0 *μ*g/ml). Neither 5 *μ*g/ml nor 10 *μ*g/ml aescin administration showed obviously protective effects, which means it worked in a dose-dependent manner. Furthermore, aescin at a concentration of 50 *μ*g/ml resulted in a greater protective effect with less cytotoxicity after OGD and reperfusion. Values are mean ± SE. ^∗^*P* < 0.05 vs. sham (no OGD); ^#^*P* < 0.05 vs. no aescin treatment (0 *μ*g/ml). *n* = 6 per group.

To determine whether aescin treatment can provide protective effects against I/R injury, we applied the OGD and reperfusion model to simulate the situation of neuronal I/R injury in vitro. Moreover, we treated primary cultured neurons with different concentrations of aescin after OGD and reperfusion to identify the appropriate dose. As displayed in Figure 1(a), primary neurons treated with aescin showed a less injury than the OGD group. Treatment with 25 *μ*g/ml, 50 *μ*g/ml, and 100 *μ*g/ml aescin significantly reduced the OGD and reperfusion-induced LDH release compared to the control group, while the concentration of 5 *μ*g/ml and 10 *μ*g/ml did not (Figure 1(b)). We supposed that aescin worked in a dose-dependent manner. Furthermore, at a concentration of 50 *μ*g/ml, aescin treatment decreased the LDH-releasing ratio with less cytotoxicity after OGD and reperfusion (*n* = 6). Thus, we selected the dose of 50 *μ*g/ml aescin for subsequent experiments.

### 3.2. Aescin Treatment Increased the Phosphorylation of the PRAS40/mTOR Signaling Pathway after OGD and Reperfusion

**Figure 2:**
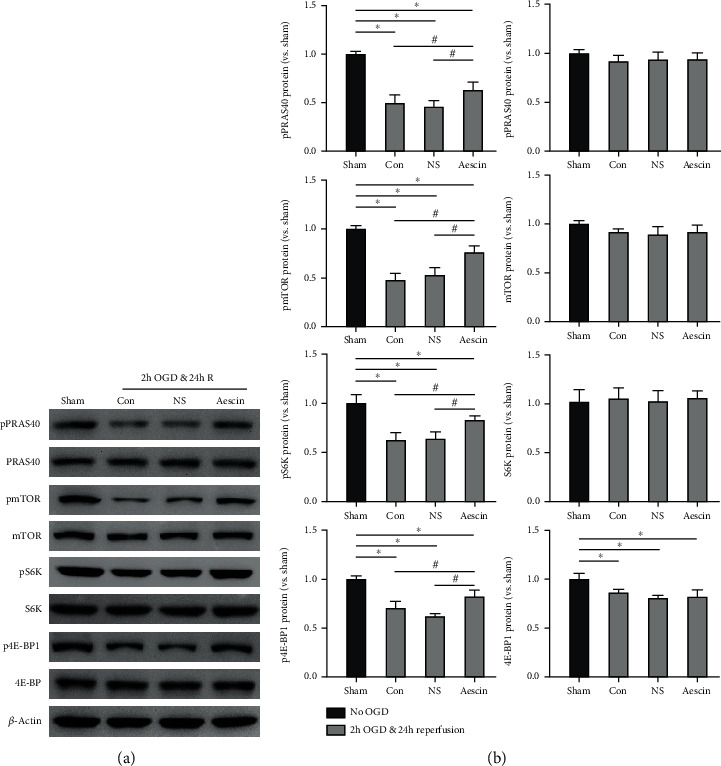
Aescin treatments upregulated the phosphorylation of PRAS40, mTOR, S6K, and 4E-BP1 in neurons after OGD and reperfusion. (a) Representative protein bands of critical molecules in the PRAS40/mTOR pathways were measured by western blot. The results showed the protein bands of phosphorylated and nonphosphorylated PRAS40, mTOR, S6K, and 4E-BP1 after 2 hours of OGD followed by 24 hours of reperfusion with 50 *μ*g/ml aescin treatment or without aescin treatment in primary cultured neurons. Protein bands of the sham group (no OGD) were also displayed. *β*-Actin was used as a control to ensure equal protein loading. (b) The bar graphs represented the relative quantified protein levels of the phosphorylated and nonphosphorylated PRAS40, mTOR, S6K, and 4E-BP1, respectively. All results are given as means ± SEM. ^∗^*P* < 0.05 vs. sham (no OGD); ^#^*P* < 0.05 vs. aescin treatment (50 *μ*g/ml). *n* = 6 per group. Sham group: no OGD samples; Con group: OGD/R samples without treatment; NS group: OGD/R samples treated with normal saline; aescin group: OGD/R samples treated with aescin.

We examined the expression of phosphorylated and nonphosphorylated PRAS40, mTOR, and the downstream proteins to confirm whether the PRAS40/mTOR pathway was involved in the protective effects of aescin. The western blot results showed that the phosphorylation level of PRAS40 and mTOR was downregulated after OGD and reperfusion, as well as S6 kinase (S6K) and 4E-binding protein 1 (4E-BP1), the downstream of mTOR signaling pathway (Figure 2(a)). Aescin treatment could partly reverse this situation and significantly promote the phosphorylation of the PRAS40, mTOR, S6K, and 4E-BP1 compared with the OGD group (Figure 2(b)) (*n* = 6). However, aescin treatment does not alter the total expression level of these proteins. These results suggest that the aescin treatment may protect neurons against OGD and reperfusion-induced injury by increasing the phosphorylation of the PRAS40/mTOR signaling pathway.

### 3.3. PRAS40 Overexpression Protected Neuron against OGD and Reperfusion Injury and Promoted the Phosphorylation of the mTOR Signaling Pathway

**Figure 3:**
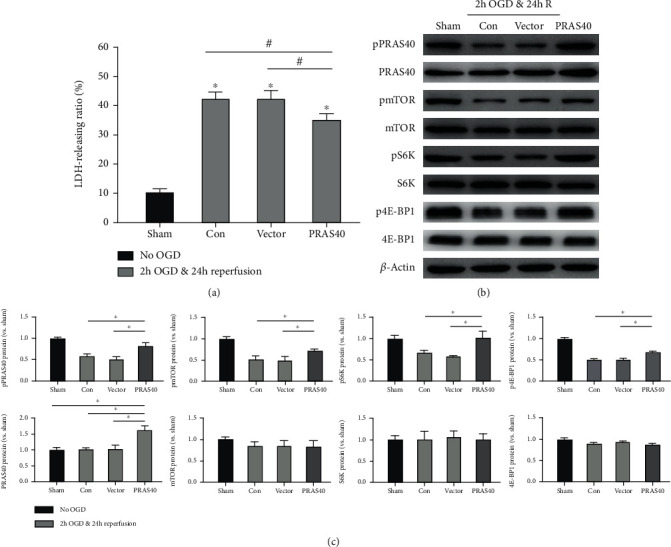
Neuroprotective effects of PRAS40 overexpression after OGD/R in vivo. (a) The effects of PRAS40 overexpression on neuronal death were measured by LDH release as before. Primary cultured neurons were transfected with lentivirus containing PRAS40; controls were cultures transfected with GFP vectors. Then, they were subjected to 2 hours of OGD 96 hours after transfection. LDH release was measured 24 hours post-OGD and reperfusion. All data after OGD was normalized to the values of the sham group. (b) Representative results showed protein bands in the PRAS40/mTOR pathways, including phosphorylated and total protein expression, as measured by western blot. (c) The bar graphs showed the quantified protein levels of pPRAS40, PRAS40, pmTOR, mTOR, pS6K, S6K, p4E-BP1, and 4E-BP1, respectively. All results are given as means ± SEM. ^∗^*P* < 0.05 vs. sham (no OGD); ^#^*P* < 0.05 vs. PRAS40; ^†^*P* < 0.05 vs PRAS40. *n* = 6 per group. Sham group: no OGD samples; Con group: OGD/R samples without treatment; vector group: OGD/R samples treated with empty vector; PRAS40 KD group: OGD/R samples treated with PRAS40 lentivirus.

To determine the role of PRAS40 in I/R injury, lentivirus overexpressing PRAS40 was transferred to primary cultured neurons. The injury degree of a neuron was evaluated by the LDH-releasing ratio as before. Compared with the control vector group, PRAS40 gene transfer reduced the LDH-releasing ratio (*n* = 6, *P* < 0.05), which indicated that PRAS40 overexpression could ameliorate the injury degree of neurons (Figure 3(a)). The phosphorylation of the mTOR signaling pathway was also examined by western blot. After OGD and reperfusion, PRAS40 overexpression could increase the level of phosphorylated mTOR, S6K, and 4E-BP1 (Figures 3(b) and 3(c)), while the phosphorylation of them was downregulated in the control vector group. The results indicated that PRAS40 overexpression may improve the survival rate of neurons via increasing the phosphorylation of the mTOR signaling pathway.

### 3.4. PRAS40 Knockdown Resulted in Severer I/R Injury and Less Phosphorylation of the mTOR Signaling Pathway

**Figure 4:**
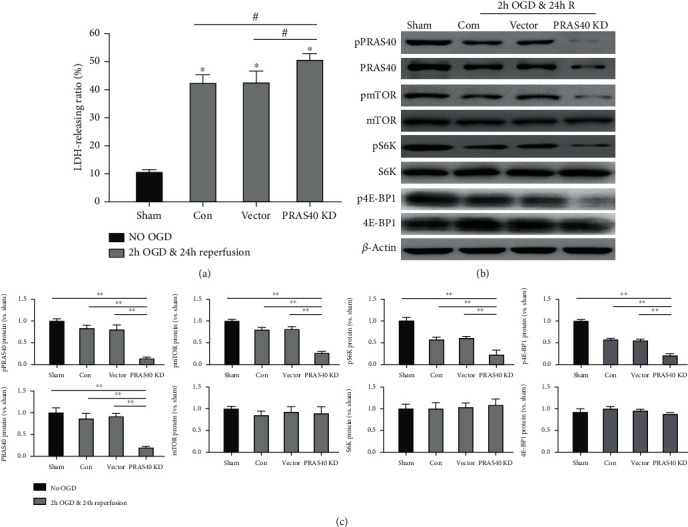
The effects of PRAS40 knockdown after OGD and reperfusion in vivo. (a) Primary cultured neurons were transfected with lentivirus containing PRAS40 shRNA 4 days before experiments; the control group was transfected with vectors expressing GFP. Then, they were subjected to the same OGD and reperfusion procedures. The LDH release represented neuronal death was measured by the same method. (b) PRAS40 knockdown (PRAS40 KD) did inhibit the expression of pmTOR, pS6K, and p4E-BP1 obviously. Representative western blot results showed the protein bands in the PRAS40/mTOR pathways, including phosphorylated and nonphosphorylated protein levels. (c) The bar graphs showed the relatively quantified protein levels of pPRAS40, PRAS40, pmTOR, mTOR, pS6K, S6K, p4E-BP1, and 4E-BP1, respectively. All results are given as means ± SEM. ^∗^*P* < 0.05 vs. sham (no OGD); ^#^*P* < 0.05 vs. PRAS40 KD; ^∗∗^*P* < 0.01 vs. PRAS40 KD. *n* = 6 per group. Sham group: no OGD samples; Con group: OGD/R samples without treatment; vector group: OGD/R samples treated with empty vector; PRAS40 KD group: OGD/R samples treated with PRAS40 shRNA.

After demonstrating the effects of PRAS40 overexpression, lentivirus containing PRAS40 shRNA was used to consolidate the effects of PRAS40 knockdown after OGD and reperfusion. The LDH-releasing ratio significantly increased in the PRAS40 knockdown group than the control vector group after OGD and reperfusion (*n* = 6, *P* < 0.05) (Figure 4(a)). These results indicated that PRAS40 knockdown could aggravate the injury of neurons after the OGD and reperfusion. Nonphosphorylated and phosphorylated proteins in the mTOR signaling pathway were measured in the same method. As shown in Figures 4(b) and 4(c), the phosphorylation of mTOR, S6K, and 4E-BP1 in the PRAS40 knockdown group was significantly lower than that in the control vector group after OGD and reperfusion. This may be the underlying mechanism of an increased neuronal death rate.

### 3.5. PRAS40/mTOR Signaling Pathway Is Essential for the Protective Effects of Aescin against I/R Injury

**Figure 5:**
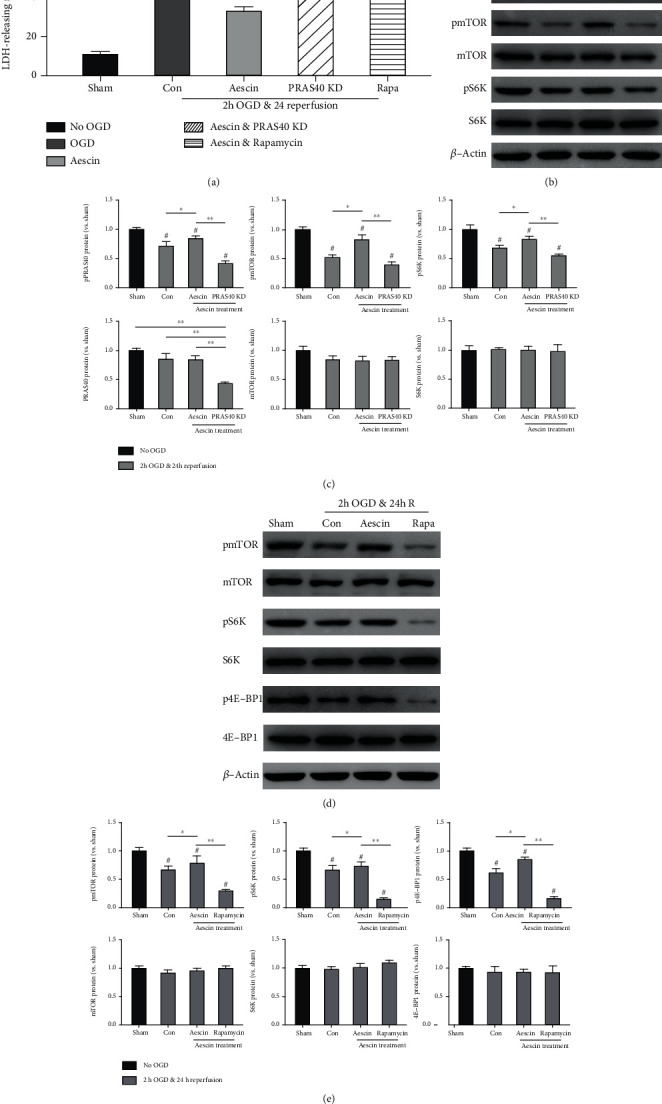
PRAS40 knockdown and rapamycin treatment undermined the protective effects of aescin against I/R injury. (a) Vectors containing PRAS40 shRNA were transferred to neurons 96 hours before OGD; the cultures were maintained without OGD or with 2 hours of OGD, followed by 24 hours of simulated reperfusion with aescin treatment; rapamycin was used at the beginning of simulated reperfusion after OGD. LDH release was measured and compared with the groups without PRAS40 shRNA or rapamycin treatment, and all results of LDH release after OGD were normalized to the values of the sham group. PRAS40 knockdown (PRAS40 KD) and rapamycin treatment reversed the decrease of LDH release induced by aescin treatments which indicated that they undermine the protective effects of the aescin treatments in primary cultured neurons after OGD and reperfusion. (b) Representative images showed the protein bands of phosphorylated PRAS40, mTOR, and S6K expression with or without aescin treatment after OGD and reperfusion. Bands of proteins of the group without OGD were displayed. *β*-Actin was used as a reference of equal protein loading. (c) The bar graphs represented the relative optical densities of phosphorylated and total PRAS40, mTOR, and S6K from the protein bands shown above. (d) Representative protein bands showed phosphorylated and total mTOR, S6K, and 4E-BP1 with or without rapamycin treatment followed by OGD and reperfusion measured by western blot; *β*-actin was used as a control to ensure equal protein loading. (e) The bar graphs represented the relative expression of phosphorylated mTOR, S6K, and 4E-BP1 from the protein bands shown above. All results are given as means ± SEM. ^∗^*P* < 0.05, respectively, vs. sham (no OGD) or Con (OGD); ^#^*P* < 0.05 vs. aescin; ^∗∗^*P* < 0.01, respectively, vs. PRAS40 KD or rapamycin. *n* = 6 per group. Sham group: no OGD samples; Con group: OGD/R samples without treatment; aescin group: OGD/R samples treated with aescin; KD group: OGD/R samples treated with aescin and PRAS40 shRNA; Rapa group: OGD/R samples treated with aescin and rapamycin.

Studies mentioned above have suggested that aescin could reduce the OGD and reperfusion-induced injury in primary cultured neuron cells and activate PRAS40 through phosphorylation. To further confirm the mechanism of aescin, we applied aescin accompanied with PRAS40 shRNA to examine whether the alteration of PRAS40 expression could undermine the neuroprotective effects of aescin. According to our results, aescin treatment decreased LDH release after OGD and reperfusion. In contrast, PRAS40 knockdown significantly increased the level of LDH release even with aescin treatment, which means it abolished the protective effects of aescin (Figure 5(a)) (*n* = 6). Moreover, the phosphorylation of proteins in the mTOR signaling pathway was inhibited because of PRAS40 knockdown (Figures 5(b) and 5(c)).

Rapamycin was given to testify whether the protective effects of aescin were correlated to the mTOR signaling pathway. The group treated simultaneously with aescin and rapamycin showed a higher LDH-releasing ratio compared with the aescin treatment group (Figure 5(a)). Western blot data showed that rapamycin could obviously block the mTOR signaling pathway, which led to a stable lower phosphorylation level of proteins in the mTOR signaling pathway (Figures 5(d) and 5(e)). These results suggested that the protective effects of aescin treatment were closely associated with the activation of the PRAS40/mTOR signaling pathway.

## 4. Discussion

Stroke has become the second leading cause of death and the most common cause of complex chronic disability worldwide [2]. In a clinic, widely use of endovascular thrombectomy and intravenous thrombolysis leads to more attention on cerebral I/R injury. Until now, there are no effective treatments for cerebral I/R injury because various pathophysiological processes such as necrosis, apoptosis, and autophagy are involved in it [23, 24].

Lots of studies have suggested that aescin could ameliorate neuronal loss, inhibit cell apoptosis, and promote microvasculation [10, 25, 26]. Aescin also protected vascular endothelial cells from injury induced by mimicked hypoxia [27]. Moreover, it has been reported that aescin promoted neurologic function recovery and reduced hippocampal damage in a transient global cerebral ischemia [28]. But limited evidence supports the application of aescin treatments against cerebral I/R injury and clarifies the possible mechanism clearly. In order to testify our hypothesis, we chose the OGD and reperfusion model in primary cultured neurons to simulate artery recanalization-induced cerebral I/R injury in vitro. As our results showed, aescin treatment obviously decreased the LDH-releasing ratio after 2 hours of OGD followed by 24 hours of reperfusion, which indicated that aescin was capable to protect neurons against OGD and reperfusion-induced injury. Meanwhile, our findings demonstrated that aescin worked in a dose-dependent way. At a dose of 50 *μ*g/ml in vitro, aescin could provide neuroprotective effects with less side effects. Beyond this concentration, the more aescin was administrated, the more cytotoxicity was presented.

PRAS40, a proline-rich Akt substrate, was identified as a regulator of the mTOR signaling pathway [29]. PRAS40 in conjunction with mTOR is closely associated with diabetes, cardiovascular diseases, and neurological diseases, such as improving insulin sensitivity [30], decreasing cardiomyocyte apoptosis after myocardial infarction [31] and neuroprotectivity after transient focal cerebral ischemia [32]. Our study suggested that aescin could significantly upregulated the phosphorylation level of PRAS40 and mTOR in primary cultured neurons after OGD and reperfusion, while the phosphorylated PRAS40 and mTOR was decreased without aescin treatment. S6K and 4E-BP1 are considered as the best-characterized downstream effectors of the mTOR signaling pathway [33], after OGD and reperfusion; the phosphorylated S6K and 4E-BP1 had the same trend with phosphorylated PRAS40 and mTOR; both of them were upregulated after aescin treatment compared with the OGD and reperfusion group.

However, the role of PRAS40 in the mTOR signaling pathway is still controversial [34]. Some of the studies stated that mTOR activity in some cell lines was inhibited by PRAS40 overexpression [35]; silencing PRAS40 would increase the phosphorylation level of the mTOR signaling pathway [36]. In contrast, other studies have determined that PRAS40 is essential to activate the mTOR signaling pathway [37] and is an important bridge linking Akt and mTOR signaling pathways [19]. In order to confirm the interaction between PRAS40 and mTOR signaling pathways in neuronal I/R injury, we used lentivirus-expressing PRAS40 to testify the role of PRAS40. It revealed that PRAS40 gene transfer promoted the phosphorylation of proteins in the mTOR signaling pathway, including mTOR, S6K, and 4E-BP1. In addition, PRAS40 overexpression reduced the release of LDH, which referred to ameliorating the I/R injury of primary cultured neurons. But the loss of PRAS40, knockdown by PRAS40 shRNA, showed an exact opposite trend. It inhibited the phosphorylation of proteins in the mTOR signaling pathway and aggravated the neuronal injury induced by OGD and reperfusion. Thus, our data support that PRAS40 is essential for mTOR activation and its downstream effectors in neuronal I/R injury.

Further experiments were performed to determine whether the protective effects of aescin was actually mediated by the PRAS40/mTOR signaling pathway. In this study, we applied aescin simultaneously with PRAS40 shRNA to investigate how PRAS40 mediated the biological function of aescin. Our results indicated that PRAS40 knockdown would lead to an increase of LDH release and undermine or even offset the protective effects of aescin against I/R injury. It appears that PRAS40 is one of the crucial biological media of aescin treatment. Furthermore, rapamycin, a specific inhibitor of mTOR, was also given to testify the role of the mTOR signaling pathway in aescin protection against I/R injury. Consistent with our previous study [17], rapamycin significantly inhibited the phosphorylation of the mTOR signaling pathway, impaired the protective effects of aescin treatment, and end up with severer neuronal I/R injury. All of these evidences strongly suggest that aescin treatment protects primary cultured neurons against I/R injury and the PRAS40/mTOR signaling pathway is necessary for the protective effects of aescin. The protective effects of aescin treatment were closely associated with the activation of the PRAS40/mTOR signaling pathway. Moreover, we verify that PRAS40 is important for the activation of the mTOR signaling pathway in cerebral I/R injury, which differs from other diseases.

For years, aescin has been used in the clinical therapy of brain trauma or intracranial hemorrhage. But the clinical application in subsequent cerebral I/R injury is rarely reported. This study demonstrates the protective effects of aescin treatment against the neuronal I/R injury and reveals that the PRAS40/mTOR signaling pathway may be the underlying mechanism of aescin protection. However, this study has potential limitations. All of these results only provide in vitro evidences for the protective effects of aescin.

It is still unclear whether or not aescin protects I/R injury in vivo and warrants investigation in the future study by using PRAS40 overexpression/knockout mice.

## 5. Conclusions

Aescin treatment is capable to protect primary cultured neurons against I/R injury in vitro. The PRAS40/mTOR signaling pathway is necessary for the protective effects of aescin against neuronal I/R injury in vitro. The results might contribute to the potential clinical application of aescin and provide a potential therapeutic target on I/R injury after endovascular thrombectomy or intravenous thrombolysis.

## Figures and Tables

**Table 1:** Antibodies, concentrations, and manufacturers used.

Antibodies	Source	Dilutions	Manufacturer	Catalog#
Phospho-mTOR (Ser2448)	Rabbit	1 : 1000	CST	2971
mTOR	Rabbit	1 : 1000	CST	2983
Phospho-S6K p70 (Ser371)	Rabbit	1 : 1000	CST	9208
S6K p70	Rabbit	1 : 1000	CST	9202
Phospho-PRAS40 (Thr246)	Rabbit	1 : 1000	CST	13175
PRAS40	Rabbit	1 : 1000	CST	2691
Phospho-4E-BP1 (Thr37/46)	Rabbit	1 : 1000	CST	2855
4E-BP1	Rabbit	1 : 1000	CST	9644
*β*-Actin	Mouse	1 : 5000	Sigma	A-5441

## Data Availability

The raw data supporting the findings of this study are available from the corresponding author on reasonable request.

## References

[1] Rosamond W., Flegal K., Furie K. (2008). Heart disease and stroke statistics--2008 update: a report from the American Heart Association Statistics Committee and Stroke Statistics Subcommittee. *Circulation*.

[2] Lopez A. D., Mathers C. D., Ezzati M., Jamison D. T., Murray C. J. L. (2006). Global and regional burden of disease and risk factors, 2001: systematic analysis of population health data. *The Lancet*.

[3] Flynn R. W. V., MacWalter R. S. M., Doney A. S. F. (2008). The cost of cerebral ischaemia. *Neuropharmacology*.

[4] Andrabi S. S., Parvez S., Tabassum H. (2017). Progesterone induces neuroprotection following reperfusion-promoted mitochondrial dysfunction after focal cerebral ischemia in rats. *Disease Models & Mechanisms*.

[5] Amirzargar M. A., Yaghubi F., Hosseinipanah M. (2017). Anti-inflammatory effects of valproic acid in a rat model of renal ischemia/reperfusion injury: alteration in cytokine profile. *Inflammation*.

[6] Jin J. K., Blackwood E. A., Azizi K. (2017). ATF6 decreases myocardial ischemia/reperfusion damage and links ER stress and oxidative stress signaling pathways in the heart. *Circulation Research*.

[7] Sciarretta S., Volpe M., Sadoshima J. (2014). Mammalian target of rapamycin signaling in cardiac physiology and disease. *Circulation Research*.

[8] Araki S., Izumiya Y., Rokutanda T. (2015). Sirt7 contributes to myocardial tissue repair by maintaining transforming growth factor-*β* signaling pathway. *Circulation*.

[9] Wang T., Fu F., Zhang L., Han B., Zhu M., Zhang X. (2009). Effects of escin on acute inflammation and the immune system in mice. *Pharmacological Reports*.

[10] Hu X. M., Zhang Y., Zeng F. D. (2004). Effects of beta-aescin on apoptosis induced by transient focal cerebral ischemia in rats. *Acta Pharmacologica Sinica*.

[11] Fu F., Hou Y., Jiang W., Wang R., Liu K. (2005). Escin: inhibiting inflammation and promoting gastrointestinal transit to attenuate formation of postoperative adhesions. *World Journal of Surgery*.

[12] Hu X. M., Zhang Y., Zeng F. D. (2004). Effects of sodium beta-aescin on expression of adhesion molecules and migration of neutrophils after middle cerebral artery occlusion in rats. *Acta Pharmacologica Sinica*.

[13] Wang T., Jiang N., Han B. (2011). Escin attenuates cerebral edema induced by acute omethoate poisoning. *Toxicology Mechanisms and Methods*.

[14] Li B., Wu G. L., Dai W. (2018). Aescin-induced reactive oxygen species play a pro-survival role in human cancer cells via ATM/AMPK/ULK1-mediated autophagy. *Acta Pharmacologica Sinica*.

[15] Sun Y., Jiang X., Pan R. (2020). Escins isolated from Aesculus chinensis Bge. promote the autophagic degradation of mutant huntingtin and inhibit its induced apoptosis in HT22 cells. *Frontiers in Pharmacology*.

[16] Wang K., Jiang Y., Wang W., Ma J., Chen M. (2015). Escin activates AKT-Nrf2 signaling to protect retinal pigment epithelium cells from oxidative stress. *Biochemical and Biophysical Research Communications*.

[17] Gao X., Chen W., Li J. (2018). The protective effect of alpha-lipoic acid against brain ischemia and reperfusion injury via mTOR signaling pathway in rats. *Neuroscience Letters*.

[18] Xie R., Li X., Ling Y. (2012). Alpha-lipoic acid pre- and post-treatments provide protection against in vitro ischemia-reperfusion injury in cerebral endothelial cells via Akt/mTOR signaling. *Brain Research*.

[19] Xiong X., Xie R., Zhang H. (2014). PRAS40 plays a pivotal role in protecting against stroke by linking the Akt and mTOR pathways. *Neurobiology of Disease*.

[20] Baldassarro V. A., Marchesini A., Giardino L., Calzà L. (2017). Vulnerability of primary neurons derived from Tg2576 Alzheimer mice to oxygen and glucose deprivation: role of intraneuronal amyloid-*β* accumulation and astrocytes. *Disease Models & Mechanisms*.

[21] Sun B., Ou H., Ren F. (2018). Propofol inhibited autophagy through Ca2+/CaMKK*β*/AMPK/mTOR pathway in OGD/R-induced neuron injury. *Molecular Medicine*.

[22] Yang H., Gao X. J., Li Y. J. (2019). Minocycline reduces intracerebral hemorrhage-induced white matter injury in piglets. *CNS Neuroscience & Therapeutics*.

[23] Xiao L. Y., Kan W. M. (2017). p53 modulates the effect of ribosomal protein S6 kinase1 (S6K1) on cisplatin toxicity in chronic myeloid leukemia cells. *Pharmacological Research*.

[24] Fenton T. R., Gout I. T. (2011). Functions and regulation of the 70kDa ribosomal S6 kinases. *The International Journal of Biochemistry & Cell Biology*.

[25] Sirtori C. R. (2001). Aescin: pharmacology, pharmacokinetics and therapeutic profile. *Pharmacological Research*.

[26] Cheng P., Kuang F., Ju G. (2016). Aescin reduces oxidative stress and provides neuroprotection in experimental traumatic spinal cord injury. *Free Radical Biology & Medicine*.

[27] Montopoli M., Froldi G., Comelli M., Prosdocimi M., Caparrotta L. (2007). Aescin protection of human vascular endothelial cells exposed to cobalt chloride mimicked hypoxia and inflammatory stimuli. *Planta Medica*.

[28] Zhang L., Fu F., Zhang X., Zhu M., Wang T., Fan H. (2010). Escin attenuates cognitive deficits and hippocampal injury after transient global cerebral ischemia in mice via regulating certain inflammatory genes. *Neurochemistry International*.

[29] Malla R., Wang Y., Chan W. K., Tiwari A. K., Faridi J. S. (2015). Genetic ablation of PRAS40 improves glucose homeostasis via linking the AKT and mTOR pathways. *Biochemical Pharmacology*.

[30] Völkers M., Doroudgar S., Nguyen N. (2014). PRAS40 prevents development of diabetic cardiomyopathy and improves hepatic insulin sensitivity in obesity. *EMBO Molecular Medicine*.

[31] Völkers M., Konstandin M. H., Doroudgar S. (2013). Mechanistic target of rapamycin complex 2 protects the heart from ischemic damage. *Circulation*.

[32] Saito A., Hayashi T., Okuno S., Nishi T., Chan P. H. (2006). Modulation of proline-rich akt substrate survival signaling pathways by oxidative stress in mouse brains after transient focal cerebral ischemia. *Stroke*.

[33] Sancak Y., Thoreen C. C., Peterson T. R. (2007). PRAS40 is an insulin-regulated inhibitor of the mTORC1 protein kinase. *Molecular Cell*.

[34] Chong Z. Z. (2016). Targeting PRAS40 for multiple diseases. *Drug Discovery Today*.

[35] Rapley J., Oshiro N., Ortiz-Vega S., Avruch J. (2011). The mechanism of insulin-stimulated 4E-BP protein binding to mammalian target of rapamycin (mTOR) complex 1 and its contribution to mTOR complex 1 signaling. *The Journal of Biological Chemistry*.

[36] Chong Z. Z., Shang Y. C., Wang S., Maiese K. (2012). PRAS40 is an integral regulatory component of erythropoietin mTOR signaling and cytoprotection. *PLoS One*.

[37] Wiza C., Nascimento E. B. M., Ouwens D. M. (2012). Role of PRAS40 in Akt and mTOR signaling in health and disease. *American Journal of Physiology. Endocrinology and Metabolism*.

